# Designing Complex Tapestries with Photography‐Inspired Manipulation of Self‐Organized Thin‐Films

**DOI:** 10.1002/advs.202401625

**Published:** 2024-04-06

**Authors:** C. T. van Campenhout, M. H. Bistervels, J. Rietveld, H. Schoenmaker, M. Kamp, W. L. Noorduin

**Affiliations:** ^1^ AMOLF Science Park 104 Amsterdam 1098XG The Netherlands; ^2^ Van 't Hoff Institute for Molecular Sciences University of Amsterdam Science Park 904 Amsterdam 1090 GD The Netherlands

**Keywords:** chemical conversion, layered compounds, photographic processing, reaction‐diffusion, self‐assembly

## Abstract

Thin‐films patterned with complex motifs are of fundamental interest because of their advanced optical, mechanical and electronic properties, but fabrication of these materials remains challenging. Self‐organization strategies, such as immersion controlled reaction‐diffusion patterning, have shown great potential for production of patterned thin‐films. However, the autonomous nature of such processes limits controllable pattern customizability and complexity. Here, it is demonstrated that photography inspired manipulation processes can overcome this limitation to create highly‐complex tapestries of micropatterned films (MPF's). Inspired by classical photographic processes, MPF's are developed, bleached, exposed, fixed, and contoured into user‐defined shapes and photographic toning reactions are used to convert the chemical composition MPF's, while preserving the original stripe patterns. By applying principles of composite photography, highly complex tapestries composed of multiple MPF layers are designed, where each layer can be individually manipulated into a specific shape and composition. By overcoming fundamental limitations, this synergistic approach broadens the design possibilities of reaction‐diffusion processes, furthering the potential of self‐organization strategies for the development of complex materials.

## Graphical abstract

Here, a strategy is introduced to shape and convert self‐organized micropatterned thin‐films (MPF's) into complex motifs using photography inspired manipulation processes. By tapping into a century's worth of photographic research, contouring and chemical conversion of patterns is performed. Inspired by composite photography, stacks of MPF layers are designed, where the shape and composition of each layer is controlled, to create complex tapestries.

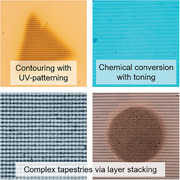

## 1 Introduction

**Figure 1:**
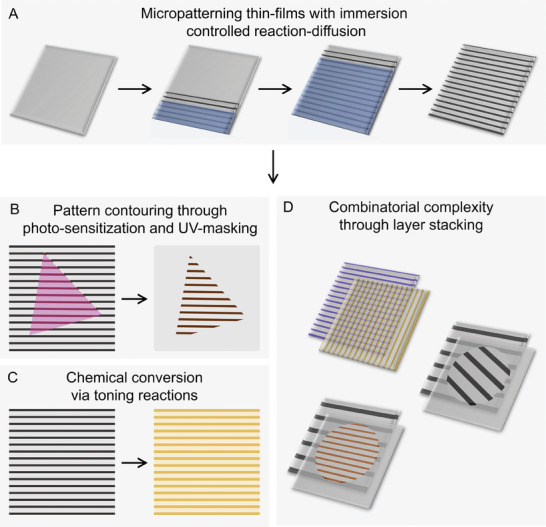
Photography inspired manipulation processes to customize micropatterned films. A) Immersion controlled reaction‐diffusion patterning to yield MPF's; by steadily immersing a gel thin‐film into a solution with silver ions, we induce periodic precipitation of silver nanoparticles arranged in uniform stripes. B) Contouring of MPF's with photo‐sensitization and subsequent UV‐masking. C) Chemical conversion of MPF's by applying toning reactions. D) Combinatorial complexity through stacking of multiple MPF layers allows for complex tapestry design.

Spatial organization of materials in thin films can enhance and exceed the properties that would be achievable by simply the sum of their parts.^[^ ^1^, ^2^, ^3^, ^4^, ^5^ ^]^ These patterned materials possess many interesting properties and can for instance be used as diffraction gratings for optical devices, anti‐reflection coatings for radiative cooling applications, or as nanoscopic capacitors for data storage.^[^ ^6^, ^7^, ^8^, ^9^, ^10^, ^11^, ^12^ ^]^ To realize such remarkable properties, it is essential to order the right material at the right place. Already, top‐down manufacturing can yield highly complex and user‐defined patterns, but the requirement of high precision equipment and specialty chemicals, makes the process oftentimes costly and difficult to scale. From this perspective, bottom‐up self‐organization strategies are promising to produce complex materials in economically and environmentally friendly ways.^[^ ^13^, ^14^, ^15^, ^16^, ^17^, ^18^, ^19^, ^20^, ^21^ ^]^ Specifically, reaction‐diffusion processes are interesting, because they use a delicate interplay between reaction kinetics and diffusion to autonomously order materials into patterns.^[^ ^22^, ^23^, ^24^, ^25^ ^]^ Recently, we have developed a self‐organization strategy based on reaction‐diffusion to structure silver nanoparticles into well‐defined micropatterned thin films (MPF's, **Figure** **1**A).^[^ ^26^ ^]^ By steadily immersing a gel thin‐film into a solution with silver ions, we induce precipitation of silver nanoparticles in the gel. These nanoparticles order in highly uniform stripes, regulated by a reaction‐diffusion process. Here, a balance between the rates of immersion and diffusion provides both hands‐on tunability of the line spacing, as well as error‐correction against defects for scalable production of uniform patterns.

However, using reaction‐diffusion patterning limits the customizability of patterns that can be generated: 1) the interplay between long‐range chemical transport and short‐range reaction‐diffusion results in large‐scale uniformity, restricting control in local shaping where patterning occurs;^[^ ^27^ ^]^ 2) the necessary delicate interplay between reaction and diffusion requires specific combinations of materials, limiting the choice in chemical composition of patterns;^[^ ^28^ ^]^ 3) the autonomous nature of reaction‐diffusion induces specific types of patterns–typically stripes and dots, thus preventing the formation of complex and user‐defined patterns.^[^ ^29^ ^]^ Hence, top‐down and bottom‐up methods offer complementary advantages and limitations. This analysis highlights that the development of synergistic combinations of such methods may offer the new routes for the synthesis of patterned thin films using both hands‐off self‐organization and hands‐on customization.

The key insight is that photographic manipulation processes have the potential to overcome the limitations of self‐organized reaction‐diffusion patterning while also offering customization. For more than a century, research into classical silver halide photography has enabled production of high‐resolution images.^[^ ^30^, ^31^ ^]^ Specifically, darkroom techniques such as development, bleaching, and toning have been developed to change the exposure, contrast and color of photographic images.^[^ ^32^ ^]^ These kind of photographic manipulation processes enable fabrication of artificial images such as composites. We hypothesize that these photographic techniques can be applied for top‐down customization of self‐organized patterns. Both photographic plates and MPF's consist of silver particles embedded in thin gelatin films, suggesting that adaptation of photographic manipulation for MPF customization is feasible. However, there are also distinct differences between photographic plates and MPF's: for classical photography, microscopic silver particles are dispersed randomly throughout the entire film, whereas for MPF's nanoscopic silver particles are ordered in distinct stripes (Figure S1, Supporting Information).^[^ ^33^, ^34^ ^]^ So, despite the chemical similarities, these structural differences render the adaptation of photographic manipulation for MPF customization non‐trivial.

In this work, we introduce photography inspired manipulation processes for the customization of self‐organized thin films. Analogous to classical photographic processes, we develop, bleach, expose, fix and contour MPF's into user‐defined complex shapes (Figure 1B). We show that photographic toning processes can convert MPF's, thereby expanding the choice of chemical composition for these patterns (Figure 1C). We demonstrate the versatility and customizability of our strategy by applying principles of composition photography to produce stacks of MPF layers, each with independently designed shape and chemical composition, to produce highly complex patterned tapestries (Figure 1D).

## 2 Results and Discussion

**Figure 2:**
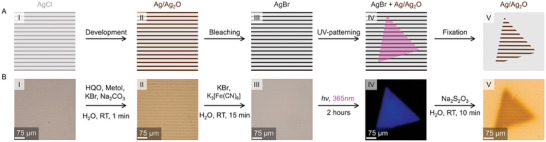
Photo‐sensitization to customize MPF's, A) schematic, and B) experimental. The process starts with self‐organized micropatterned silver film containing silver chloride, which is poorly visible due to low contrast (I). These MPF's are developed, which converts silver chloride into silver/silver oxide, making the pattern visible by increasing contrast and ensuring compatibility with subsequent processes (II). Then a photo‐sensitization step is performed, converting all silver/silver oxide particles into light sensitive silver bromide (III). By illuminating a triangular section of this sensitized film with UV light (365 nm), a part of the MPF is converted from silver bromide back to silver/silver oxide (IV). All silver bromide is then solubilized using a fixation step, to yield an MPF with stripes only in the illuminated area (V).

To demonstrate the proof‐of‐principle, we first produce uniform MPF's and render them compatible with photographic processes. To produce uniform MPF's, we steadily immerse a gelatin thin film (0.3 mm) into a solution of silver nitrate (0.4 M), yielding silver chloride (AgCl) nanoparticles arranged in tunable uniform microscopic (*d* = 10 − 30 µm) stripe patterns (**Figure** **2**A,B I).^[^ ^26^, ^35^ ^]^ To make these patterns compatible with subsequent photographic processes and to increase the contrast of the pattern, we immerse the MPF into a photographic developer solution that reduces and converts all (AgCl) particles to Ag/Ag_2_O. Analysis with optical microscopy reveals a stark increase in contrast, and, importantly, shows that the original stripe pattern and spacing are preserved (Figure 2A,B II).

We contour this MPF using ultraviolet (UV) light. For this, we first make the MPF light sensitive by performing a bleaching procedure. In such a procedure, silver/silver oxide (Ag/Ag_2_O) particles convert into light sensitive silver bromide (AgBr) by immersing the MPF in an aqueous solution of potassium bromide and potassium ferricyanide (K_3_[Fe(CN)_6_]) for 15 min at room temperature (Figure 2A,B III). The AgBr MPF is locally illuminated with UV light (365 nm),^[^ ^36^ ^]^ using a triangular photo‐mask (Figure 2A,B IV, see Supporting Information for experimental details). In the illuminated region, AgBr is converted into Ag/Ag_2_O. Because the particles in an MPF are at least an order of magnitude smaller than those found in photographic films, the particles are much less light sensitive. To counteract this insensitivity, we illuminate with a strong UV‐LED (535 µWmm^−2^) for much longer (>2 h) than is common in traditional photography. Subsequently, we reveal the photo‐pattern by applying a fixation process that removes unreacted AgBr, which is present in areas that have not been illuminated with UV‐light. For this, the MPF is immersed in an aqueous solution of sodium thiosulfate (Na_2_S_2_O_3_) and washed with water, which solubilizes and removes all unreacted AgBr (Figure 2A,BV). Although slight blurring is observed around the edges—attributed to imperfect photomasking, we find that the original stripe pattern is preserved and contoured precisely in the shape of the applied photo‐mask, demonstrating the concept of photography inspired manipulation for customizing MPF shapes.

The success of the photo‐sensitization process suggests that photographic manipulation processes may be applied to customize MPF's in different ways. Specifically, many reactions have been developed to change the color or longevity of photographs. These so‐called toning reactions work by converting the chemical composition of photographs, while retaining the original image. We realize that these photographic toning reactions may enable the chemical conversion of MPF's, and thus hold the potential to disentangle the shape and composition of MPF's.

We investigate this potential of photographic toning recipes by first forming an MPF, which we then develop and tone using various toning recipes. We characterize the pattern and chemical composition before and after each processing step using optical microscopy and energy dispersive x‐ray spectroscopy analysis (EDXA). We find that the insulating and flexible nature of the gelatin matrix hinders direct EDXA of the chemical composition of the pattern. To overcome this limitation, we analyze the chemical composition by first liquefying the gel, followed by centrifugation to extract the particles. The extracted particles are then re‐dispersed and dropcasted for EDXA (see Supporting Information for experimental details and for SEM micrographs of the measured particles).

**Figure 3:**
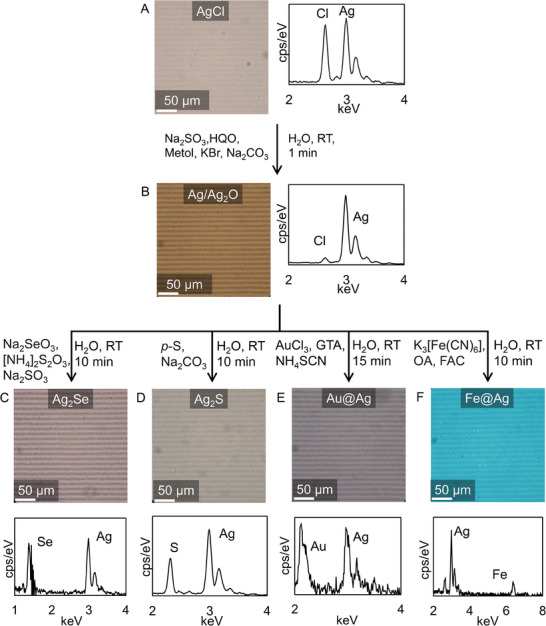
Chemical conversion through photographic toning reactions A) Optical microscopy image of a non‐processed MPF and EDXA, revealing that the pattern consists of silver chloride particles. B) Optical microscopy image of a developed MPF and EDXA showing that additional silver is precipitated on top of existing silver chloride. Optical microscopy image and EDXA confirming conversion of developed MPF after different toning reactions to C) silver selenide, D) silver sulfide, E) gold‐silver core‐shell particles, and F) iron‐silver core‐shell particles. Note that all microscopy images were shot at identical contrast and magnification, without additional color correction, to show color changes after toning.

EDXA reveals that initially the MPF indeed consists of AgCl particles, where likely some metallic silver is present because of light exposure (atomic ratio Ag:Cl = 1.24:1, **Figure** **3**A). We realize that by using a development step, we can convert these AgCl particles into Ag/Ag_2_O, to ensure compatibility with toning processes. For this, we immerse the MPF in a commonly used aqueous developer solution containing sodium sulfite, hydroquinone, metol, potassium bromide, and sodium carbonate for one minute. EDXA shows that nearly all AgCl is converted to Ag/Ag_2_O (atomic ratio Ag:Cl = 22:1, Figure 3B), with preservation of the stripe pattern.

We explore how these developed MPF's can be converted into a range of different chemical compositions. Specifically, to demonstrate the proof‐of‐principle, we investigate both anionic and cationic toning conversions. For anionic conversions, we apply two commonly used toning processes: selenium and sulfide toning. For selenium toning, we immerse a developed MPF in an aqueous solution of sodium selenite, ammonium thiosulfate, and sodium sulfite (commercially available Ilford Harman Selenium toner) for 10 min at room temperature to form silver selenide (Ag_2_Se), as confirmed by EDXA (Figure 3C, atomic ratio Ag:Se = 1.68:1). For sulfide toning, we immerse a developed MPF in an aqueous solution of polysulfide and sodium carbonate at room temperature for 10 min to produce silver sulfide (Ag_2_S) particles, again confirmed by EDXA (Figure 3D, atomic ratio Ag:S = 1.75:1). Importantly, in both cases the original stripe pattern and microscale spacing is preserved, showing pattern‐preserving chemical conversion of MPF's using anionic toning processes.

To demonstrate cationic conversions, we apply two commonly used cationic toning reactions: gold toning and Prussian blue (iron) toning. For gold toning, we immerse a developed MPF in an aqueous solution of gold (III) chloride and ammonium thiocyanate (commercially available Bergger Goldtoner). This toning solution causes the gelatin matrix to swell. We prevent this undesired swelling by adding a small amount of the cross‐linker glutaraldehyde (0.1%) to the toning solution. During this toning process, gold grows on pre‐existing Ag/Ag_2_O particles and forms a coating, yielding gold‐silver (Au@Ag) core‐shell particles,^[^ ^37^ ^]^ consistent with EDXA of the resulting MPF (atomic ratio Au:Ag = 0.42:1). With Prussian blue toning, a similar coating of iron can be produced.^[^ ^38^ ^]^ For this, a developed MPF is immersed in an aqueous solution of potassium ferricyanide, oleic acid, and ferric ammonium citrate in water for 10 min at room temperature, yielding iron‐silver (Fe@Ag) core‐shell particles, demonstrated by a vibrant blue color and the appearance of a distinct iron peak at 6.4 keV in EDXA (molar ratio Fe:Ag = 0.51:1). These results demonstrate that both anionic and cationic conversions can be realized using photographic toning reactions, to customize the chemical composition of MPF's with preservation of the original stripe pattern.

Thus, by combining self‐organized pattern formation with photographic manipulation processes we gain three levels of control over MPF's: 1) spacing of tunable stripe patterns through immersion controlled reaction‐diffusion; 2) contouring using photo‐sensitization and UV‐masks; 3) converting chemical composition via toning reactions. These three levels of control can be combined into higher‐order complex motifs.

We realize that in traditional photographic manipulation processes, multiple layers of photographic negatives can be stacked to create composite images. Akin to such composite photographs, we can produce higher‐order complex motifs by stacking layers of MPF's. This opens a combinatorial and versatile design space: by manipulating each individual layer with UV‐contouring and chemically conversion, we can create highly complex user‐defined tapestries.

**Figure 4:**
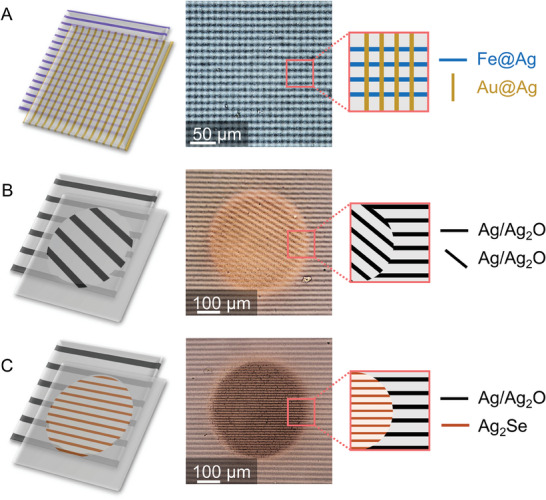
Inspired by composite photography, stacking of MPF layers provides a combinatorial complex design space. A) Stacking two spatially identical MPF's of different chemical composition produces a grid where horizontal stripes consist of Fe@Ag and vertical lines consist of Au@Ag. B) Stacking two UV‐patterned MPF's, where the first layer contains stripes only in the positive image of a circle and the second layer only in the negative image of a circle, generates a complex tapestry. C) Stacking two MPF's with non‐identical pattern spacing, varying chemical composition, and different UV‐patterns demonstrates the full combinatorially complex design space.

We demonstrate this design potential with three examples. First, by stacking two MPF layers of different chemical composition at 90°, we produce square patterns, where the horizontal lines contain Fe@Ag and the vertical lines contain Au@Ag (**Figure** **4**A). Second, by stacking MPF layers contoured with different UV‐masks, we enable the formation of complex motifs with arbitrary selected patterning directions. Here, we demonstrate this by contouring a negative circular image into one MPF, and a positive circular image into another. By stacking these opposite motifs, we obtain a tapestry where the orientation of the pattern is different in and outside of the circle (Figure 4B). Third, to demonstrate an even more refined level of complexity, we design a tapestry composed of two MPF layers, each with different line spacing, different shape and different chemical composition. For the first layer, we produce a stripe pattern with line spacing 22 µm and contour a negative circle. For the second layer, we produce a stripe pattern with line spacing 10 µm and contour a positive circle, followed by selenium toning. Stacking these two layers yields a highly complex tapestry, with user‐defined pattern spacing, shape and chemical composition (Figure 4C).

## 3 Conclusion

In summary, we introduce photography inspired manipulation processes to design complex tapestries of self‐organized thin‐films. Intriguingly, there exists an historical connection between self‐organized patterning and photography: in the late 19th century, the German scientist R. E. Liesegang pioneered photography techniques, and serendipitously discovered self‐ organized patterns.^[^ ^39^ ^]^ These so‐called Liesegang patterns are at the core of the immersion controlled reaction‐diffusion process used to create MPF's. Now, 130 years later, we reunite Liesegang patterns with their photographic roots, to design complex thin‐film tapestries. We realize that with immersion controlled reaction‐diffusion we can create MPF's that are chemically very similar to classical silver halide photographs. Leveraging these similarities unlocks the complete toolbox of darkroom techniques for user‐defined manipulation of MPF's.^[^ ^32^ ^]^ Using photography inspired techniques, we can independently control the shape via photo‐sensitization combined with UV‐masking, and alter the chemical composition through conversion with toning reactions. Akin to composite photographs, we stack multiple individually manipulated MPF layers to create complex tapestries.

The power of our approach is that it combines the strengths of self‐organization and photographic manipulation. By harnessing the autonomy of self‐organization and exploiting the automated mass‐production of photographic materials, this synergistic approach grants user‐control across length‐scales: from microscopic control over shape and chemical composition to macroscopic scale‐up.

The success of this strategy is surprising: darkroom techniques have been developed for photographs, not MPF's. Nevertheless, we demonstrate that these century old techniques can be readily adapted for recently developed pattern formation processes. Building upon these results, we foresee that other interesting photographic reactions and processes can be extended to fields outside of photography. Specifically, we project exciting opportunities for the field of ion‐exchange, where insights from photographic toning processes could inspire the design of new conversion reactions.

Additionally, we foresee new functionalities that exploit the unique combination of tunable long‐range ordering with local customization. For instance, already layer stacking allows the formation of Moiré patterns,^[^ ^26^ ^]^ which combined with local customization could now be designed for information processing, sensing, and advanced optical devices. Furthermore, if self‐organized patterns are scaled down one order of magnitude, these structures could exhibit structural coloration. In combination with the local customizability of photography inspired manipulation processes, these structures would allow the fabrication of structurally colored images.

To summarize, we have shown a synergistic approach combining hands‐off self‐organization and hands‐on manipulation for the synthesis of complex thin‐film tapestries. We envisage that top‐down customization of self‐organized patterns using photography inspired manipulation processes will lead to exciting opportunities for the generation of complex patterns with advanced functionalities.

## Conflict of Interest

The authors declare no conflict of interest.

## Supporting information

Supporting Information

## Data Availability

The data that support the findings of this study are available from the corresponding author upon reasonable request.

## References

[1] P. Ball , The self‐made tapestry: pattern formation in nature, Oxford University Press, Inc., Oxford 1999.

[2] U. G. Wegst , H. Bai , E. Saiz , A. P. Tomsia , R. O. Ritchie , Nat. Mater. 2015, 14, 23. 25344782 10.1038/nmat4089

[3] M. Eder , S. Amini , P. Fratzl , Science 2018, 362, 543. 30385570 10.1126/science.aat8297

[4] J. Huang , X. Wang , Z. L. Wang , Nano Lett. 2006, 6, 2325. 17034105 10.1021/nl061851t

[5] Y. Helfman Cohen , Y. Reich , S. Greenberg , J. Mech. Des. 2014, 136, 111108.

[6] Y. Gu , Y. Zhang , J. Lin , H. Zhao , H. Ma , H. Yao , M. Kang , B. Fu , S. Liu , Colloids Surf. A: Physicochem. Eng. Asp. 2023, 679, 132569.

[7] E. Akerboom , T. Veeken , C. Hecker , J. Van De Groep , A. Polman , ACS Photonics 2022, 9, 3831. 36573162 10.1021/acsphotonics.2c01389 PMC9782778

[8] W. Lee , H. Han , A. Lotnyk , M. A. Schubert , S. Senz , M. Alexe , D. Hesse , S. Baik , U. Gösele , Nat. Nanotechnol. 2008, 3, 402. 18654563 10.1038/nnano.2008.161

[9] M. R. Begley , D. S. Gianola , T. R. Ray , Science 2019, 364, eaav4299. 31249029 10.1126/science.aav4299

[10] L. Wågberg , J. Erlandsson , Adv. Mater. 2021, 33, 2001474. 10.1002/adma.202001474 PMC11468756 32767441

[11] Y. Wang , I. Fedin , H. Zhang , D. V. Talapin , Science 2017, 357, 385. 28751606 10.1126/science.aan2958

[12] A. R. Studart , Adv. Mater. 2012, 24, 5024. 22791358 10.1002/adma.201201471

[13] G. M. Whitesides , B. Grzybowski , Science 2002, 295, 2418. 11923529 10.1126/science.1070821

[14] D. Philp , J. F. Stoddart , Angew. Chem., Int. Ed. Engl. 1996, 35, 1154.

[15] L. Cera , C. A. Schalley , Adv. Mater. 2018, 30, 1707029. 10.1002/adma.201707029 29931699

[16] P. Knoll , O. Steinbock , Isr. J. Chem. 2018, 58, 682.

[17] W. L. Noorduin , A. Grinthal , L. Mahadevan , J. Aizenberg , Science 2013, 340, 832. 23687041 10.1126/science.1234621

[18] Z. Bao , M. R. Weatherspoon , S. Shian , Y. Cai , P. D. Graham , S. M. Allan , G. Ahmad , M. B. Dickerson , B. C. Church , Z. Kang , H. W. Abernathy III , C. J. Summers , M. Liu , K. H. Sandhage , Nature 2007, 446, 172. 17344850 10.1038/nature05570

[19] N. Vogel , M. Retsch , C.‐A. Fustin , A. Del Campo , U. Jonas , Chem. Rev. 2015, 115, 6265. 26098223 10.1021/cr400081d

[20] J. Zhang , J. Yan , S. Granick , Angew. Chem. 2016, 128, 5252. 10.1002/anie.201509978 27010594

[21] M. Odziomek , F. Thorimbert , C. Boissiere , G. L. Drisko , S. Parola , C. Sanchez , M. Faustini , Adv. Mater. 2022, 34, 2204489. 10.1002/adma.202204489 35797893

[22] A. M. Turing , Phil. Trans. R. Soc. London. Series B, Biol. Sci. 1952, 237, 37.

[23] M. C. Cross , P. C. Hohenberg , Rev. Mod. Phys. 1993, 65, 851.

[24] B. A. Grzybowski , K. J. Bishop , C. J. Campbell , M. Fialkowski , S. K. Smoukov , Soft Matter 2005, 1, 114.

[25] B. A. Grzybowski , Chemistry in motion: reaction‐diffusion systems for micro‐and nanotechnology, John Wiley & Sons, New York 2009.

[26] C. T. van Campenhout , H. Schoenmaker , M. van Hecke , W. L. Noorduin , Adv. Mater. 2023, 35, 2305191. 10.1002/adma.202305191 37471706

[27] C. T. van Campenhout , D. N. Ten Napel , M. van Hecke , W. L. Noorduin , Proc. Nat. Acad. Sci. 2022, 119, e2123156119. 36122212 10.1073/pnas.2123156119 PMC9522343

[28] H. Nabika , M. Itatani , I. Lagzi , Langmuir 2019, 36, 481. 10.1021/acs.langmuir.9b03018 31774294

[29] J. Boissonade , E. Dulos , P. De Kepper , in Chemical waves and patterns, Springer, Berlin, Heidelberg 1995, pp. 221–268.

[30] J. Hamilton , Adv. Phys. 1988, 37, 359.

[31] R. Hirsch , Seizing the light: a social & aesthetic history of photography, Taylor & Francis, Milton Park 2017.

[32] S. Anchell , The Darkroom Cookbook, Routledge, Milton Park 2016.

[33] V. P. Oleshko , Industrial Applications Of Electron Microscopy, 2002, 51.

[34] B. D. Guenther , D. Steel , Encyclopedia of modern optics, Academic Press, Cambridge 2018.

[35] R. M. Walliser , R. Tóth , I. Lagzi , D. Mathys , L. Marot , A. Braun , C. E. Housecroft , E. C. Constable , RSC Adv. 2016, 6, 28388.

[36] M. H. Bistervels , N. T. Hoogendoorn , M. Kamp , H. Schoenmaker , A. M. Brouwer , W. L. Noorduin , Nanoscale 2024, 16, 2310. 38230748 10.1039/d3nr05828j PMC10832358

[37] P. Ellis , Gold Bull. 1975, 8, 7.

[38] T. Rudman , The Photographer's Toning Book: The Definitive Guide, Amphoto Books, New York 2003.

[39] R. E. Liesegang , Naturwiss. Wochenschr 1896, 11, 353.

