# Supplementary material for: Designing Complex Tapestries with Photography‐Inspired Manipulation of Self‐Organized Thin‐Films
Source: Adv Sci (Weinh). 2024 Apr 6;11(25):2401625. doi: 10.1002/advs.202401625 (PMC11220642; doi:10.1002/advs.202401625)
Supplement: Supplementary file 1 — Supporting Information [file ADVS-11-2401625-s001.pdf]

ADVANCED SCIENCE
Open Access

Supporting Information

for *Adv. Sci.*, DOI 10.1002/advs.202401625

Designing Complex Tapestries with Photography-Inspired Manipulation of Self-Organized Thin-Films

*C. T. van Campenhout, M. H. Bistervels, J. Rietveld, H. Schoenmaker, M. Kamp and W. L. Noorduin**

Supporting Information

Designing complex tapestries with photography-inspired manipulation of self-organized thin-films

*Christiaan T. van Campenhout, Marloes H. Bistervels, Jeppe Rietveld, Hinco Schoenmaker, Marko Kamp, Willem L. Noorduin**

This document contains the following supporting information:

- Materials and Methods
    1. Self-Organization Process
    2. Photographic Manipulation Recipes
    3. SEM and EDX analysis
        Figure S1
        Figure S2
    4. UV patterning
- References

Materials and Methods

All chemicals were used without additional purification. Gelatin was purchased from Sigma-Aldrich and was type A from porcine skin (roughly 300 g Bloom). 3-[hydroxy(polyethyleneoxy)propyl] heptamethyltrisiloxane (CAS: 67674-67-3) was purchased from Gelest.

1. **Self-Organization Process**

Micropatterned films are made using a previously developed reaction-diffusion driven, immersion controlled patterning process (R-DIP). For details on this process, see [1]. In short, a thin hydrogel film is cast onto a glass substrate. This film is then slowly immersed in reagent, which diffuses into the hydrogel thereby triggering a reaction-diffusion process that forms periodic precipitation stripes.

Here, we produce microscopic patterns of silver chloride. First, gelatin (1.0 g), 3-[hydroxy(polyethyleneoxy)propyl] heptamethyltrisiloxane (15 μL) and ammonia (25% in water, 40 μL) are added to DI water (9 mL). This mixture is heated to 60° C to dissolve the gelatin, after which 0.3 mm thick gels are cast. These gels are then immersed (1.5 μm/s) in a silver nitrate solution (0.4M in water). Note that chloride ions are already present in type A gelatin, and are not added separately.

2. Photographic Manipulation Recipes

Developer (Kodak D-19)[2]

- Potassium bromide 50 mg
- Sodium Sulfite 0.90 g
- Hydroquinone 80 mg
- Metol 20 mg
- Sodium carbonate 0.45 g
- Water 8.5 mL

Dissolve at 52 °C, use at room temperature for 1 – 5 minutes.

Bleacher (Print Rehalogenating Bleach)[2]

- Potassium bromide 80 mg
- Potassium ferricyanide 120 mg
- Water 9,8 mL

Use at room temperature for 5 – 10 minutes.

Fixer (Plain Hypo) [2]

- Sodium thiosulfate 4.8 g
- Water 20 mL

Fix at room temperature for 10 minutes.

Sulfide toner (Kodak T-8)[2]

- Polysulfide 75 mg
- Sodium carbonate 25 mg
- Water 10 mL

Use at room temperature for 5 – 10 minutes.

Selenium toner

Commercially available Ilford Harman Selenium Toner, contains:

- Sodium selenite
- Ammonium thiosulfate
- Sodium sulfite
- Water

Use at room temperature for 5 – 10 minutes.

Iron toner (Kodak T-12) [2]

- Ferric ammonium citrate 20 mg
- Oxalic acid 20 mg
- Potassium ferricyanide 20 mg

Dissolve each separately in water (10 mL), mix and filter directly prior to use. Use in the dark. Precipitation occurs in light exposed aqueous solutions of ferric ammonium citrate, resulting in particles depositing on the film surface during toning if the toner is exposed to light and/or not filtered properly.

Use at room temperature for 5 – 10 minutes.

Gold toner

Commercially available Bergger Goldtoner, contains:

- Gold (III) chloride
- Ammonium thiocyanate

To prevent MPF swelling, glutaraldehyde (0.1%) was added.

Use at room temperature for 5 – 10 minutes.

3. SEM and EDX analysis

To characterize the chemical composition of the MPF's, we perform energy dispersive x-ray analysis (EDXA), using a FEI Verios 460 scanning electron microscope equipped with an Oxford X-Max^{n} energy dispersive X-ray spectrometer. Because the gelatin matrix is non-conductive, direct analysis of the particles is impossible. Via critical point drying, collapse of the gel network can be prevented, and spatial information of the particle distribution through the pattern can be obtained (Figure S1). However, because the matrix is flexible in this state, particles move away from the e-beam, making high resolution imaging and high acceleration voltages required for EDXA impractical. Therefore, we remove particles from the gelatin matrix prior to SEM/EDXA.

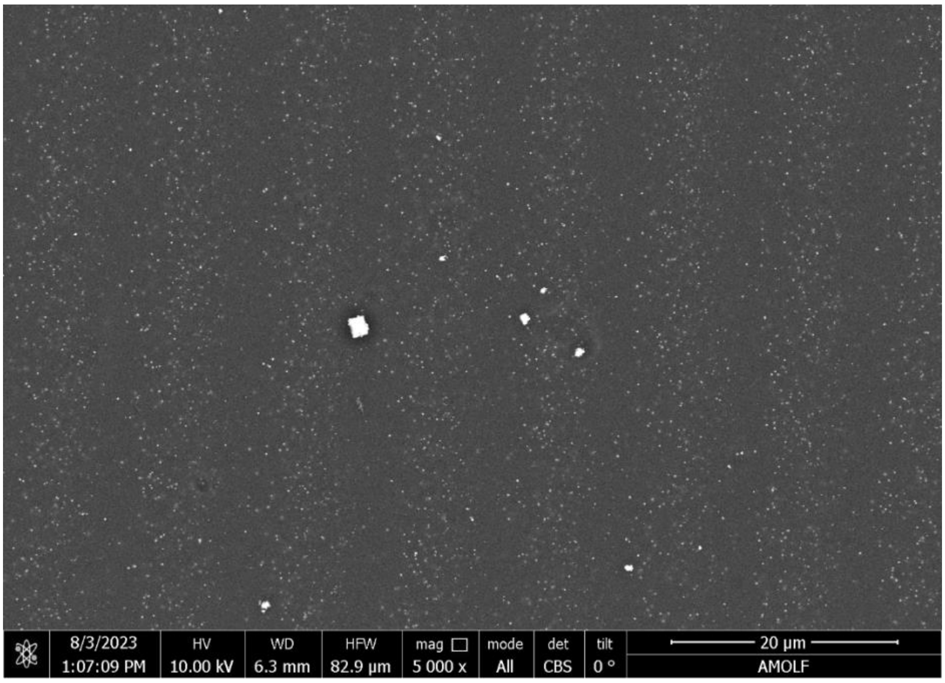

Figure S1: SEM micrograph of an MPF using a Concentric Backscatter (CBS) detector. This sample was prepared by first immersing the MPF in increasing amounts of ethanol (25% EtOH in water -> 50% EtOH in water -> 100% EtOH), followed by critical point drying using a Autosamdri 815 serie B super critical point dryer and sputter coating a 10 nm chromium layer using a Leica EM ACE600 Double sputter coater. The white dots in this micrograph are silver chloride nanoparticles (150-200 nm).

To this aim, MPF sections are placed into a 10 mL Falcon tube, to which boiling water (7 mL) is added. By vortexing directly, all gelatin is solubilized and partially hydrolyzed, such that it no longer forms a gel. By centrifuging at 6500 rpm for 1 hour the particles are separated from the gelatin solution. The supernatant is discarded and again boiling water (7 mL) is added, to remove any leftover gelatin. After again centrifuging at 6500 rpm for 1 hour, the supernatant is removed. The particles are then redispersed in 2-propanol (1 mL) and drop casted onto an aluminum substrate. Figure S2 shows the particles used for EDXA.

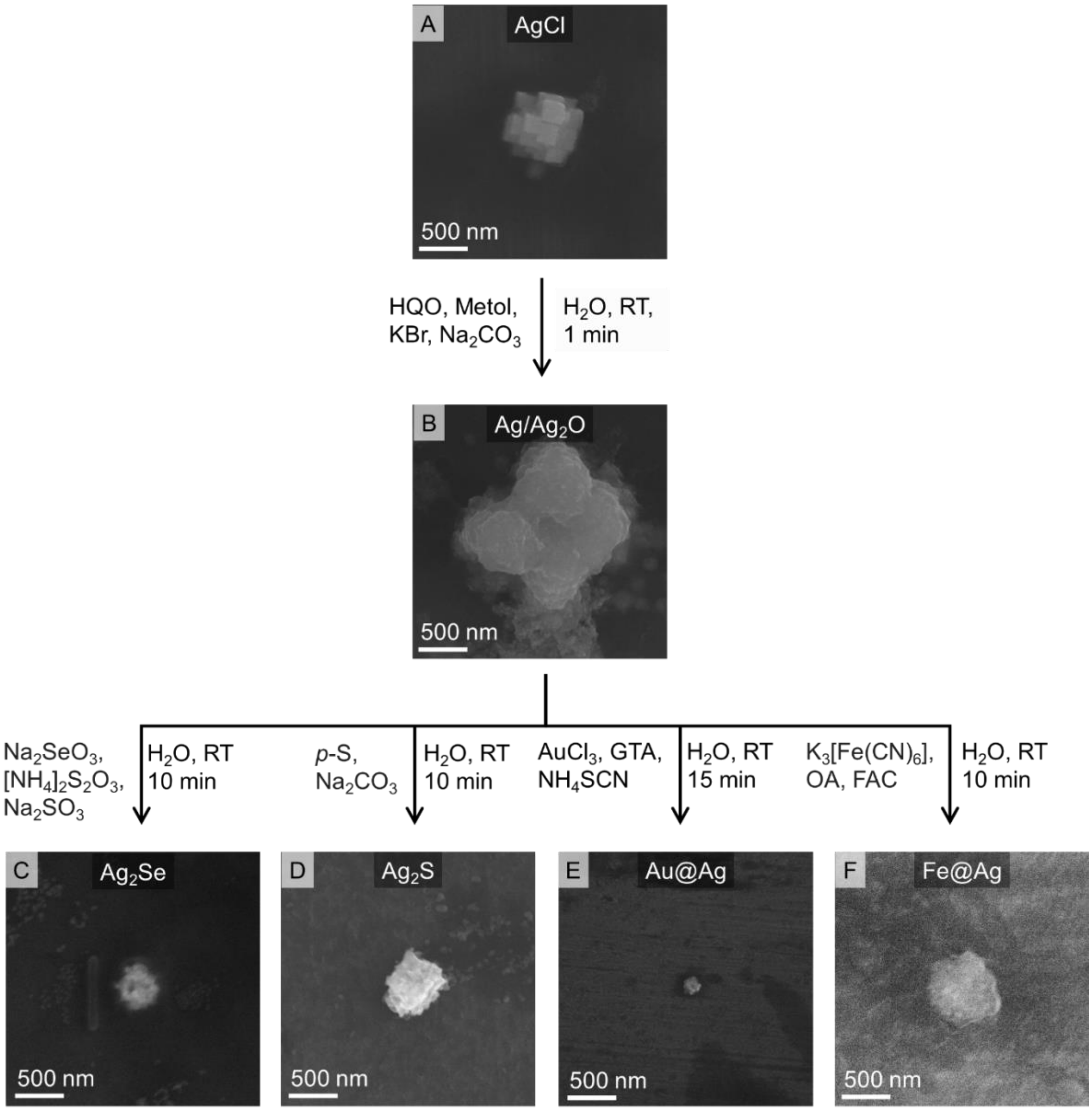

Figure S2: SEM micrographs of the particles used for the EDX analysis shown in Figure 3. (A) Silver chloride particles before any modification. (recorded at 20 kV, 100pA) (B) Silver/silver oxide particles obtained after development (recorded at 20 kV, 100pA). (C) Silver selenide particles obtained after selenium toning (recorded at 20 kV, 100pA). (D) Silver sulfide particles obtained after sulfide toning (recorded at 20 kV, 100pA). (E) Gold-silver particles obtained after gold toning (recorded at 20 kV, 100pA). (F) Iron-silver particles obtained after iron toning (recorded at 30 kV, 100pA).

4. UV patterning

UV patterning was performed using a custom-built microscopy setup. For more details on this setup, see [3]. This custom microscope contains three main sections: an irradiation parts, a sample holder stage, and an imaging part.. For the UV LED irradiation, a lens (Edmund Optics 84–337, focal length (f) = 20.0 mm) collects the light from a 365 nm mounted LED (Thorlabs M365L3, 1290 mW output power). An UV anti-reflection coated plano-convex lenses (Thorlabs LA4148-UV, f = 50.0 mm) projects custom-made photomasks onto the sample through a 10X/0.30 magnification objective (Nikon Plan Fluor). The light intensities of the UV LED is controlled by adjustments of the driving current of the drivers (Thorlabs LEDD1B). The sample holder stage is attached to a Huber CC-K6 cooling bath thermostat.

Developed MPF's were immersed in the bleaching solution (see S2) for 10 minutes and washed four times in a large excess of DI water for 10 minutes directly prior to UV-patterning. This converts all silver particles to silver bromide, which are light sensitive, and care was taken to keep samples out of direct light. Using the custom microscopy setup described above, samples were photopatterned with 365 nm UV light for >12 hours, whilst cooled at 10°C to prevent sample heating and gel decomposition, after which the MPF was removed from the sample holder and directly placed in a fixing bath (see S2). After 10 minutes the sample was washed four times with a large excess of DI water.

References

[1] C. T. van Campenhout, H. Schoenmaker, M. van Hecke, W. L. Noorduin, *Adv. Mat.* **2023,** *35*, 2305191.

[2] Anchell, Steve. *The Darkroom Cookbook*. Routledge, 2016.

[3] M. H. Bistervels, N. Hoogendoorn, M. Kamp, H. Schoenmaker, A. M. Brouwer, W. L. Noorduin, *Nanoscale,* **2024,** 16, 2310
